# Next-Generation Sequencing Characterizes the Landscape of Somatic Mutations and Pathways in Metastatic Bile Tract Carcinoma

**DOI:** 10.1155/2020/3275315

**Published:** 2020-09-04

**Authors:** Qi Li, Qifan Zhang, Xiao Cheng, Xie Weng, Mian Chen, Xiaoyun Hu, Jing Huang, Jinzhang Chen

**Affiliations:** ^1^State Key Laboratory of Organ Failure Research, Guangdong Provincial Key Laboratory of Viral Hepatitis Research, Department of Infectious Diseases, Nanfang Hospital, Southern Medical University, No. 1838, North Guangzhou Avenue, Guangzhou, Guangdong 510515, China; ^2^Department of Oncology, Nanfang Hospital, Southern Medical University, Guangzhou 510515, China; ^3^Department of Hepatobiliary Surgery, Nanfang Hospital, Southern Medical University, Guangzhou 510515, China; ^4^Cancer Center, Integrated Hospital of Traditional Chinese Medicine, Southern Medical University, Guangzhou, Guangdong 510315, China; ^5^Transplant Immunology Laboratory, Churchill Hospital, Oxford University Hospitals NHS Trust, Old Road, Headington, Oxford OX3 8HF, UK

## Abstract

**Purpose:**

Tumor metastasis remains the leading cause of cancer-related mortality in biliary tract cancer. The etiology and mechanism of bile tract carcinoma metastasis are unclear.

**Methods:**

The primary tumor and blood samples of 14 patients with biliary tract cancer were collected, followed by nucleic acid extraction and library construction. Target sequencing with 556 panel genes and WES were performed to detect the hot spot genes variations. Bioinformatics was used to comprehensively analyze the sequencing data of these samples, including the differences of tumor mutation burden and signaling pathways.

**Results:**

The results showed that the mutation frequency of *TP53* gene was the highest and the mutations of *CTNNB1*, *EPHA7*, *ARID2*, and *PIK3CA* were only found in metastatic samples. The TMB mean values of metastatic and non-metastatic groups were 12.97 and 10.38 mutations per Mb, respectively. There were significant differences in the enrichment pathways of cellular components between the tumor metastasis and non-metastatic samples.

**Conclusions:**

We identified multiple pathway differences, which helps us better understand metastatic biliary tumors and design clinical therapy for personalized medicine.

## 1. Introduction

Biliary tract carcinoma (BTC) is an invasive adenocarcinoma that originates from the epithelial cells of the biliary tract, including the intrahepatic and extrahepatic bile ducts and the gallbladder [1]. Although BTCs are considered a rare tumor, they account for about 10%–15% of all primary liver cancers [2]. Only 10% of patients have early disease and are considered candidates for surgical excision, and the recurrence rate is high despite therapeutic surgery. Most patients with locally advanced or metastatic BTCs have poor prognosis and overall survival (OS) is less than 12 months [3].

In recent years, advances in sequencing technology have promoted the application of sequencing technology in cancer research, giving scientists an in-depth understanding of these cancers at the molecular level [4, 5]. However, metastases that cause 90% of cancer deaths have been reported in prostate, breast, and colorectal cancers [6–8]. Even with a small number of metastatic cancers in tumors of the biliary tract carcinomas, only a few specific genes have been studied [9]. Some studies have shown that the process of biliary cancer tumor metastasis has complex signal pathway crossover. Neurotensin can enhance EGFR/AKT signal pathway and promote the metastasis of cholangiocarcinoma cells [10]. LncRNA-UCA1 promoted the activation of ERK/MAPK pathway by regulating the expression of miR-122 and its downstream gene mRNA CLIC1 [11]. Therefore, a better understanding of the biological and phenotypic evolution of BTCs and their molecular and genetic mechanisms during the metastatic process is crucial.

In order to further study the genetic characteristics of tumor metastasis in the biliary tract cancer system, we conducted high-depth sequencing in 14 patients with biliary tract cancer. Somatic cell mutations, tumor mutation burden, TITV, and molecular signaling pathways of mutated genes in patients with metastasis and non-metastasis were analyzed. Exploring the mechanism of metastasis in biliary tract cancer can provide effective guidance for potential clinical interventions, thus improving the long-term prognosis of patients.

## 2. Methods

### 2.1. Patient Specimen Acquisition

Primary tumor tissue and blood from 14 patients with BTCs were collected. According to the digestive system tumor classification of WHO in 2010, tumor samples were identified by hematoxylin and eosin stained slides. The tumor tissue samples included were histologically confirmed to be adenocarcinoma by two molecular pathologists, and the tumor cell content exceeded 70%. This study was conducted in accordance with the Helsinki Declaration and was approved by the first affiliated southern hospital ethics committee [12]. Prior to inclusion in the study, all patients received informed written consent and all groups participating in this study approved this work.

### 2.2. DNA Extraction and Qualification

DNA was extracted from tissue sections of tumor samples and matched with the DNA of leukocytes as germline mutation. DNeasy Blood and Tissue Kit (69504, Qiagen, Venlo, Netherlands) was used according to the manufacturer's instructions. The content of DNA was determined by Agilent Bioanalyzer (USA).

### 2.3. Target Genes Sequencing and Whole-Exome Sequencing

The targeted capture pulldown and exon-wide libraries from genomic DNA were generated through the xGen® Exome Research Panel (Integrated DNA Technologies, Inc., Illinois, USA) and the TruePrep DNA Library Prep Kit V2 for Illumina (#TD501, Vazyme, Nanjing, China). The captured libraries sequencing was performed as paired-end reads on the Illumina NoveSeq platform.

### 2.4. Variant Annotation Analysis and Visual Mapping

Split-reads were re-aligned to hg19/GRch37 using blast to exclude false positive results. We used Picard tool (http://broadinstitute.github.io/picard/command-line-overview. html#CollectHsMetrics) on the computational efficiency of WES data capture. The tool is used to filter low-quality bases, repetitive bases, bases that deviate from the target, and ends that are overlapped to read at both ends due to short inserts. This tool calculated the strictest depth distribution. If the mean bait coverage of sequencing data was more than 100, we defined the data quality control as qualified. Further local rearrangements were performed with SpeedSeq to improve the alignment of individual reads [13]. Somatic mutations identification and indels were annotated through Mutect [14] and Somatic Indel Detector software [15]. ANNOVAR [16] and Oncotator [17] annotated the variant data in use and converted it to a MAF file through the maf tool [18]. The cancer driver genes were analyzed using Intogen [19], including Oncodrive FM and Oncodrive CLUST. The landscape of top driver mutation spectrum predicted by Intogen for tumors was visualized via R Script, including mutation rate and mutation subclass/subtypes (ONCODRIVEFM *P* value ≤ 0.1).

### 2.5. Pathway Enrichment Analysis

Wayne map was used to show that there were differences in the frequency of somatic gene mutation between metastatic and non-metastatic group, because some mutation genes exist only in the metastatic group or in the non-metastatic group, while some genes were mutated in both. GO or Gene Ontology canonical pathways with R packages: clusterProfiler were used to analyze the genes that contain single nucleotide variants (SNV) or SV [20]. The calculation of *P* value was based on hypergeometric distribution, and Benjamin and Hochberg methods were used for FDR correction [21].

### 2.6. Statistical Analyses

All the correlate clinical and biological variables were employed using the SPSS Statistics 22.0 package and ggpubr package [22] in R [23] by methods of a non-parametric test (such as Welch's *t* test and Wilcoxon) when necessary.

## 3. Result

### 3.1. Patient Characteristics

To better understand the molecular mechanism of BTC metastasis, fresh-frozen tumor tissue and corresponding blood specimens from 14 BTC patients were selected for bioinformatics analysis based on stringent criteria. S08 and S09 samples were sequenced by whole exome sequencing (WES), the other 12 samples sequenced by 556 panel genes. A total of three patients had a history of alcohol consumption, and half had HBV. According to the clinical stages, there were two patients in stage I, one patient in stage IIA, three patients in stage IIIB, two patients in stage IVA, and six patients in stage IVB. In addition, nine samples including S01, S03, S04, S05, S06, S08, S09, S10, and S14 were assigned to the metastatic group, while the remaining 5 samples of S02, S07, S11, S12, and S13 were in the non-metastatic group. The detailed clinical characteristics of the patients are shown in Table 1 and Supplementary Table 1.

### 3.2. Identification of Somatic Mutations in 14 Patients with BTCs

**Figure 1:**
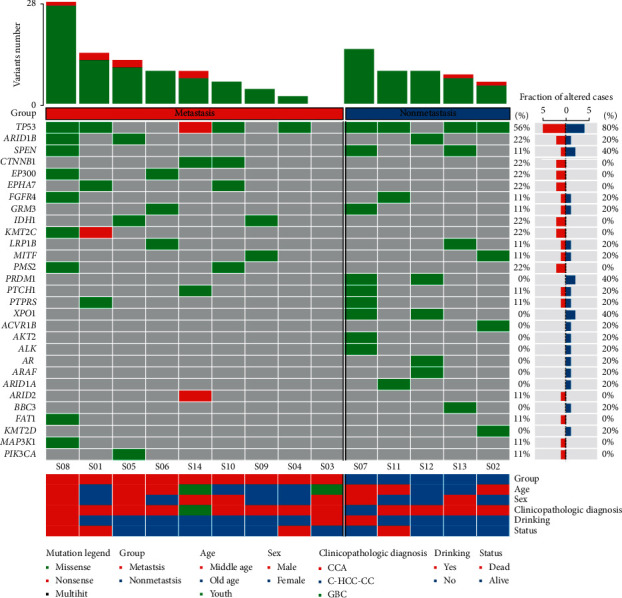
Somatic mutation in biliary tumors. The top figure shows the variation number of each sample. The middle picture shows the mutated genes and mutation types of the samples. The bottom chart shows the clinical information such as age, gender, clinical diagnosis, drinking history, survival status, etc. The red bars on the right represent the mutation frequency of somatic cell genes in patients with cholangiocarcinoma metastasis, and the blue bars represent the mutation frequency of somatic genes in patients without metastasis.

We performed NGS sequencing on DNA from 14 primary tumor tissues along with matched blood and annotated several somatic mutations using Mutect and Somatic Indel Detector: WES depth with a mean depth of 200.2X and 556 panel sequencing with a mean depth of 2559.95X Overall. We detected 119 nonsynonymous single nucleotide variations (SNVs) in all samples, including 31 nonsynonymous SNVs detected in WES and 88 nonsynonymous SNVs detected in 556 panel sequencing, which were all missense mutations (Supplementary Table 2). As shown in Figure 1, case S08 had the most SNVs, followed by case S07. We listed the top 29 genes based on the frequency of somatic mutations. Among them, *TP53* (64%) was the highest mutation gene. Missense mutation was the most common type of mutation, along with nonsense, multi hit, and so on (Figure 1). Driver genes, such as *CTNNB1* (22%), *EP300* (22%), *KMT2C* (22%), and *IDH1* (22%), were mutated only in the metastatic group, while *XPO1* (40%) mutation was found only in non-metastatic group.

**Figure 2:**
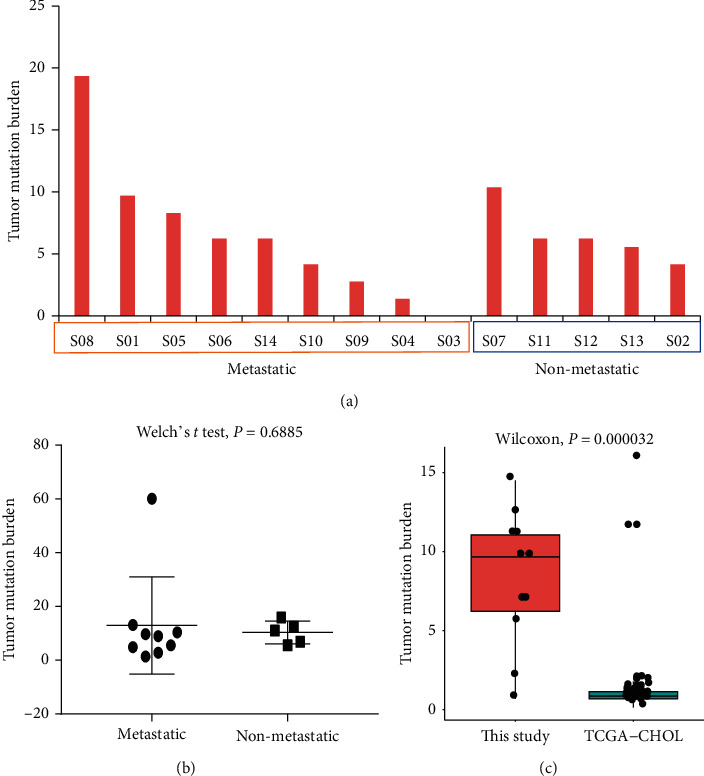
Comparative analysis of TMB difference. (a) Tumor mutation burden in metastatic and non-metastatic samples; (b) comparison of TMB between metastatic and non-metastatic samples; (c) comparison of TMB between the data in this study and the TCGA-CHOL.

We also calculated tumor mutation burden (TMB) using only somatic nonsynonymous mutations. On the whole, the TMB mean values of metastatic and non-metastatic groups were 12.97 and 10.38 mutations per Mb, respectively, and there was no significant difference between these two groups, *P* = 0.6885 (Welch's *t* test) (Figures 2(a) and 2(b)). In addition, the TMB value of this study (8.635829) was significantly higher than that of TCGA-CHOL database (1.433684), with statistical significance (Wilcoxon, *P* = 0.000032) (Figure 2(c)).

### 3.3. Characteristics of Signaling Pathways in BTCs

**Figure 3:**
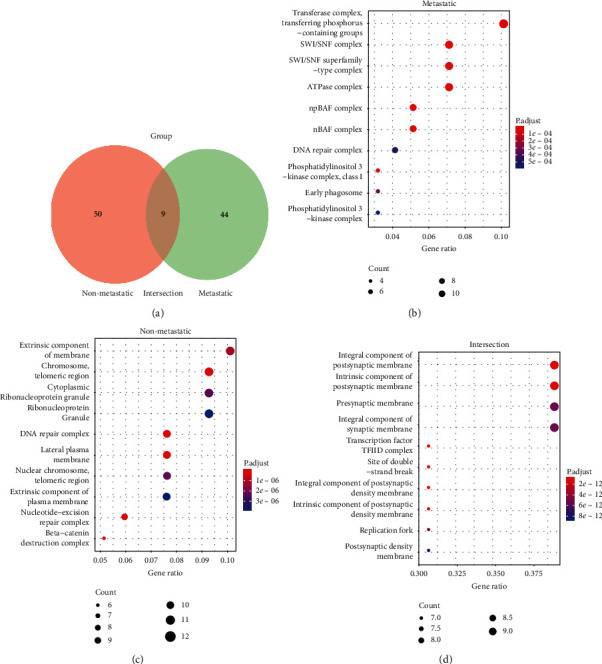
Analysis of pathways involving nonsynonymous genes in BTCs. (a) Venn diagram of gene distribution in three clusters. (b) The cellular component pathway of mutant genes in the metastatic group. (c) The cellular component pathway of mutant genes in the non-metastatic group. (d) The cellular component pathway of mutant genes in the intersection group.

In order to further characterize the functions of mutational genes and pathways involved in BTC, we used PANTHER classification system [24], an ontology-based pathway database coupled with data analysis tools. According to the metastasis of the patient, we used a Venn diagram to divide the mutant genes detected by 556 gene panel sequencing into three clusters, including metastatic genes cluster (44 genes), non-metastatic genes cluster (50 genes), and intersection genes cluster (9 genes) (Figure 3(a)).

In the metastatic cluster, the mutant somatic genes were mainly enriched in the transferase complex, SWI/SNF complex, ATPase complex, and nBAF complex pathway (Figure 3(b)). However, the altered somatic genes in the non-metastatic cluster were mainly enriched in extrinsic component of membrane, chromosome-telomeric region, cytoplasmic ribonucleoprotein granule, DNA repair complex, and lateral plasma membrane in the pathway of cellular components (Figure 3(c)). In addition, the cellular components of mutant genes in the intersection of metastatic and non-metastatic clusters were mainly in histone removal factor TFIID complex, components of synaptic membrane, intrinsic components of presynaptic membrane, postsynaptic membrane, and activity of postsynaptic membrane enzyme modulator (Figure 3(d)).

## 4. Discussion

BTC is a rare malignant tumor [25]. At the initial diagnosis stage, about 60% or 70% of cholangiocarcinomas are pathologically advanced [26], and the overall survival time is between 8 and 715 days, with an average of 302 days, which is consistent with the previous statement that OS is less than 12 months [27]. Metastatic clones can occur in the early and late stages of primary tumors [8, 28]. Better understanding of the genetic characteristics of metastatic diseases may reveal the differences between the treatment weaknesses of primary and metastatic tumors and provide insights into the biology of metastasis. The metastasis of biliary malignancies is often accompanied by mutations or changes in expression levels of multiple pathway genes, including abnormal activation of proto-oncogenes and inactivation of tumor suppressor genes. We designed 556 genes panel of tumor mutation hot spot gene and sequenced it in high depth. We also found high frequency mutated genes in tumors of the biliary system that have been reported, such as *TP53*, *ARID1B*, *CTNNB1*, *EPHA7*, *IDH1*, and so on [29–31], while *CTNNB1*, *EPHA7*, *ARID2*, and *PIK3CA* were mutated in patients with metastasis (Figure 1). It has been reported that, in intrahepatic biliary cell carcinoma, mutations in *CTNNB1* are mostly associated with alcohol intake risk factors, while mutations in *TP53* are caused by HBV risk factors [32]. Frequent mutations of *TP53* and *ARID1A* associated with chromatin remodeling and chromosomal organization may be involved in the carcinogenesis and development of intrahepatic cholangiocarcinoma [33]. We also analyzed 51 samples from the TCGA-CHOL database and found that the mutation frequency of *TP53* was only (4/51) 8%, far lower than the targeted sequencing (9/14) 62% in this study (Supplementary Figure 1). This indicates that the target gene sequencing method is more accurate in detecting target genes.

Previous studies have reported that, in univariate analysis, *TP53* and *ARID1A* were predictors of poor prognosis in cholangiocarcinoma [29]. We calculated the relationship between *TP53* mutation and overall survival time, indicating that no significant difference was found (*P* = 0.325), which may be largely due to the low sample size (Supplementary Figure 2).

Currently, there are dozens of biomarkers related to checkpoint inhibitors, among which TMB, PD-L1, and MSI/dMMR have been verified by phase III clinical trials and are widely used in clinical practice [34–36]. TMB is a biomarker for predicting PD-1/PD-L1 immune response [37, 38]. Even though it has been reported that TMB-H alone is not suitable for predicting the immunotherapy effect of solid tumor type [39], we found that there was a significant difference in TMB between this study and TCGA, but the TMB did not exceed 20 mutations per Mb (Figure 2). For different cancer types, the setting of high TMB threshold may need more clinical studies and a large number of patient information statistics. A Japanese paper suggests that TMBs are overestimated in targeted sequencing compared to WES [40]. Therefore, we compared the TMB value of BTC samples in this study with TCGA-CHOL and found that the depth of targeted sequencing was significantly higher than that of WES.

Signaling pathways are characterized by mutations in a single gene or changes in expression, which usually involve simultaneous changes in multiple pathways, such as angiogenesis and notch signaling pathway [41]. During the process of tumor metastasis, cellular components such as the transferase complex, SWI/SNF complex, ATPase complex, and nBAF complex pathway are all adjusted accordingly. SWI/SNF, including BRM or BRG-1, ATPase subunits, controls many aspects of normal cellular function [42]. Ribonucleoprotein promotes tumor metastasis by induction of genes involved in extracellular matrix, cell movement, and angiogenesis [43]. Functional recovery is promising in cancer therapy because epigenetic inhibition regulates the expression of SWI/SNF components at least in some cases. More research is needed to unblock the role of SWI/SNF in cancer and determine how it affects tumor metastasis. This is an exciting but poorly understood molecule that may play a role in causing cancer.

## 5. Conclusion

Our study identified somatic mutations, TMB in metastatic and non-metastatic groups. The GO analysis showed that the metastatic and non-metastatic groups were completely different in terms of cellular composition pathways. Although the sequencing depth of 556 panel gene was very high, the detected genes were known or have been reported, and new site mutations cannot be found. In addition, our 556 panel was designed for the locus with high mutation hotspots in the Asian population, which was suitable for the detection of mutated genes in the whole cancer species, not specifically for the cholangiocarcinoma, so the detection range of mutated genes is still limited. In addition, the small sample size and the lack of matched BTC transfer samples were also limitations of this study. In conclusion, the current findings could help identify specific pathways and hot spots that altered during metastasis and provide a direction for targeted therapy of these tumors.

## Figures and Tables

**Table 1:** Patient characteristics.

Characteristic	No. of cases	Proportion (%)
Total number	*n* = 14	

Age, years (mean)	53.28 (30–70)	

Sex		
Male	7	50.0
Female	7	50.0

Drinking history		
Drinker	3	16.7
Nondrinker	11	83.3

Metastatic	9	64.3

Non-metastatic	5	35.8

Tumor staging		
I	2	14.3
IIA	1	7.1
IIIB	3	21.4
IVA	2	14.3
IVB	6	42.9

State		
Alive	6	42.9
Dead	8	57.1

## Data Availability

All the related software and scripts are available from the corresponding author on reasonable request.

## Supplementary Materials

Supplementary Materials Supplementary figure 1: mutational landscape of somatic alterations in TCGA-CHOL database. Supplementary figure 2: Kaplan–Meier survival analysis using the log-rank test (mantel-cox test) for comparing the differences between mutant TP53 and wild-type TP53 genes. Supplementary Table 1: patient clinical information. Supplementary Table 2: NGS results of our cohort. Support files legends. Supplementary table a: variant allele frequency (VAF) of 556 genes in target sequencing data and WES data. Supplementary table b: 556 somatic mutant genes were divided into TCGA-CHOL recognized genes and unrecognized genes in this study. Supplementary table c: mutation screening of 44 transferred genes in the TCGA-CHOL dataset. Supplementary table d: list of genes involved in 556 genes panel sequencing in this study. Supplementary table e: the list of genes related to the pathways in the three clusters. Supplementary figure a: Go-CC pathway of transferred genes in TCGA database.
