# Supplementary figures and images for: Next-Generation Sequencing Characterizes the Landscape of Somatic Mutations and Pathways in Metastatic Bile Tract Carcinoma
Source: J Oncol. 2020 Sep 4;2020:3275315. doi: 10.1155/2020/3275315 (PMC7487098; doi:10.1155/2020/3275315)

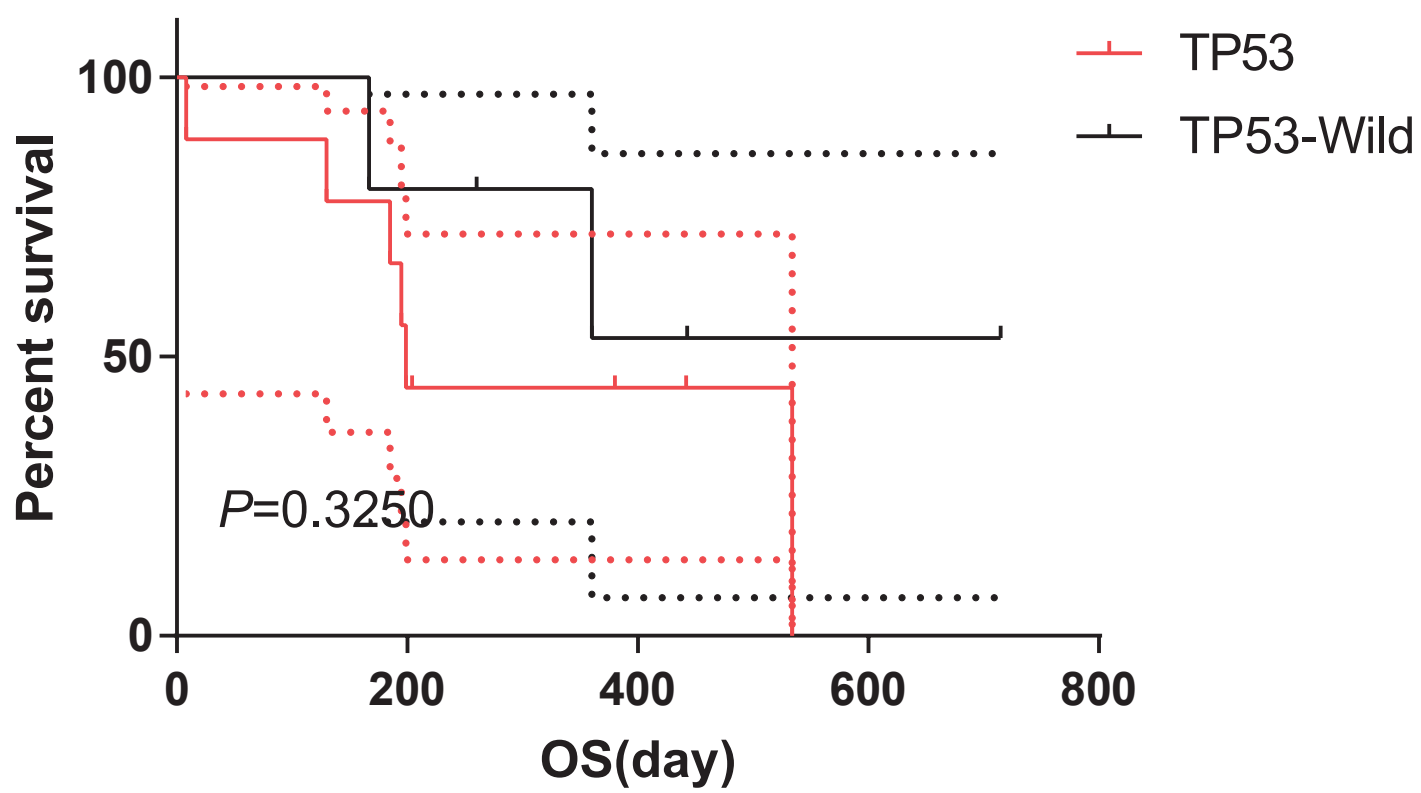

Supplement: Supplementary Materials — Supplementary figure 1: mutational landscape of somatic alterations in TCGA-CHOL database. Supplementary figure 2: Kaplan–Meier survival analysis using the log-rank test (mantel-cox test) for comparing the differences between mutant TP53 and wild-type TP53 genes. Supplementary Table 1: patient clinical information. Supplementary Table 2: NGS results of our cohort. Support files legends. Supplementary table a: variant allele frequency (VAF) of 556 genes in target sequencing data and WES data. Supplementary table b: 556 somatic mutant genes were divided into TCGA-CHOL recognized genes and unrecognized genes in this study. Supplementary table c: mutation screening of 44 transferred genes in the TCGA-CHOL dataset. Supplementary table d: list of genes involved in 556 genes panel sequencing in this study. Supplementary table e: the list of genes related to the pathways in the three clusters. Supplementary figure a: Go-CC pathway of transferred genes in TCGA database. [file 3275315.f1.zip › 3275315.f1/Supplementary figure 2.pdf]

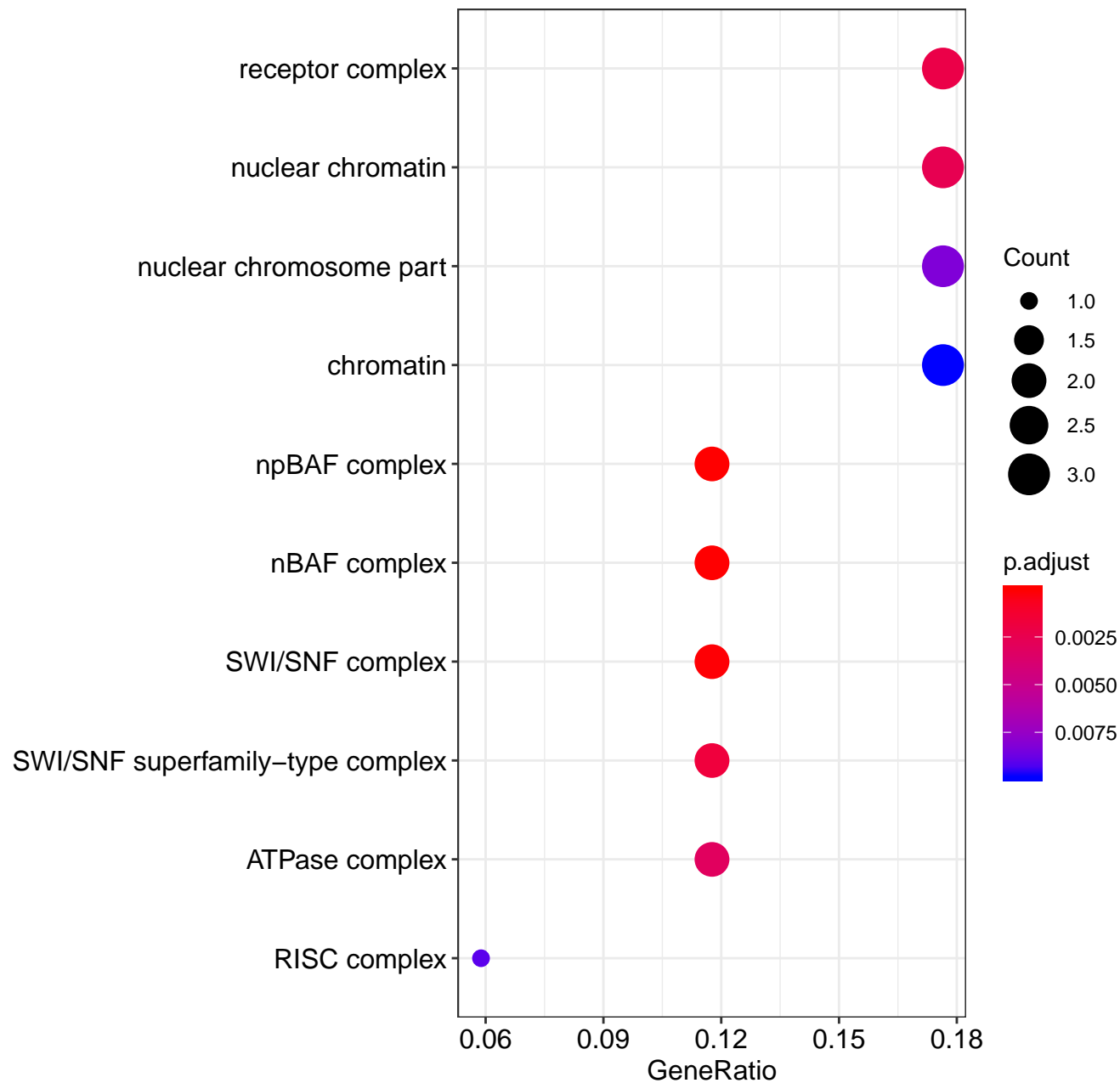

Supplement: Supplementary Materials — Supplementary figure 1: mutational landscape of somatic alterations in TCGA-CHOL database. Supplementary figure 2: Kaplan–Meier survival analysis using the log-rank test (mantel-cox test) for comparing the differences between mutant TP53 and wild-type TP53 genes. Supplementary Table 1: patient clinical information. Supplementary Table 2: NGS results of our cohort. Support files legends. Supplementary table a: variant allele frequency (VAF) of 556 genes in target sequencing data and WES data. Supplementary table b: 556 somatic mutant genes were divided into TCGA-CHOL recognized genes and unrecognized genes in this study. Supplementary table c: mutation screening of 44 transferred genes in the TCGA-CHOL dataset. Supplementary table d: list of genes involved in 556 genes panel sequencing in this study. Supplementary table e: the list of genes related to the pathways in the three clusters. Supplementary figure a: Go-CC pathway of transferred genes in TCGA database. [file 3275315.f1.zip › 3275315.f1/Supplementary figure a.pdf]
