# Supplementary material for: Next-Generation Sequencing Characterizes the Landscape of Somatic Mutations and Pathways in Metastatic Bile Tract Carcinoma
Source: J Oncol. 2020 Sep 4;2020:3275315. doi: 10.1155/2020/3275315 (PMC7487098; doi:10.1155/2020/3275315)
Supplement: Supplementary Materials — Supplementary figure 1: mutational landscape of somatic alterations in TCGA-CHOL database. Supplementary figure 2: Kaplan–Meier survival analysis using the log-rank test (mantel-cox test) for comparing the differences between mutant TP53 and wild-type TP53 genes. Supplementary Table 1: patient clinical information. Supplementary Table 2: NGS results of our cohort. Support files legends. Supplementary table a: variant allele frequency (VAF) of 556 genes in target sequencing data and WES data. Supplementary table b: 556 somatic mutant genes were divided into TCGA-CHOL recognized genes and unrecognized genes in this study. Supplementary table c: mutation screening of 44 transferred genes in the TCGA-CHOL dataset. Supplementary table d: list of genes involved in 556 genes panel sequencing in this study. Supplementary table e: the list of genes related to the pathways in the three clusters. Supplementary figure a: Go-CC pathway of transferred genes in TCGA database. [file Supplementary figure 1.pdf › 3275315.f1/Supplementary figure 1.pdf]

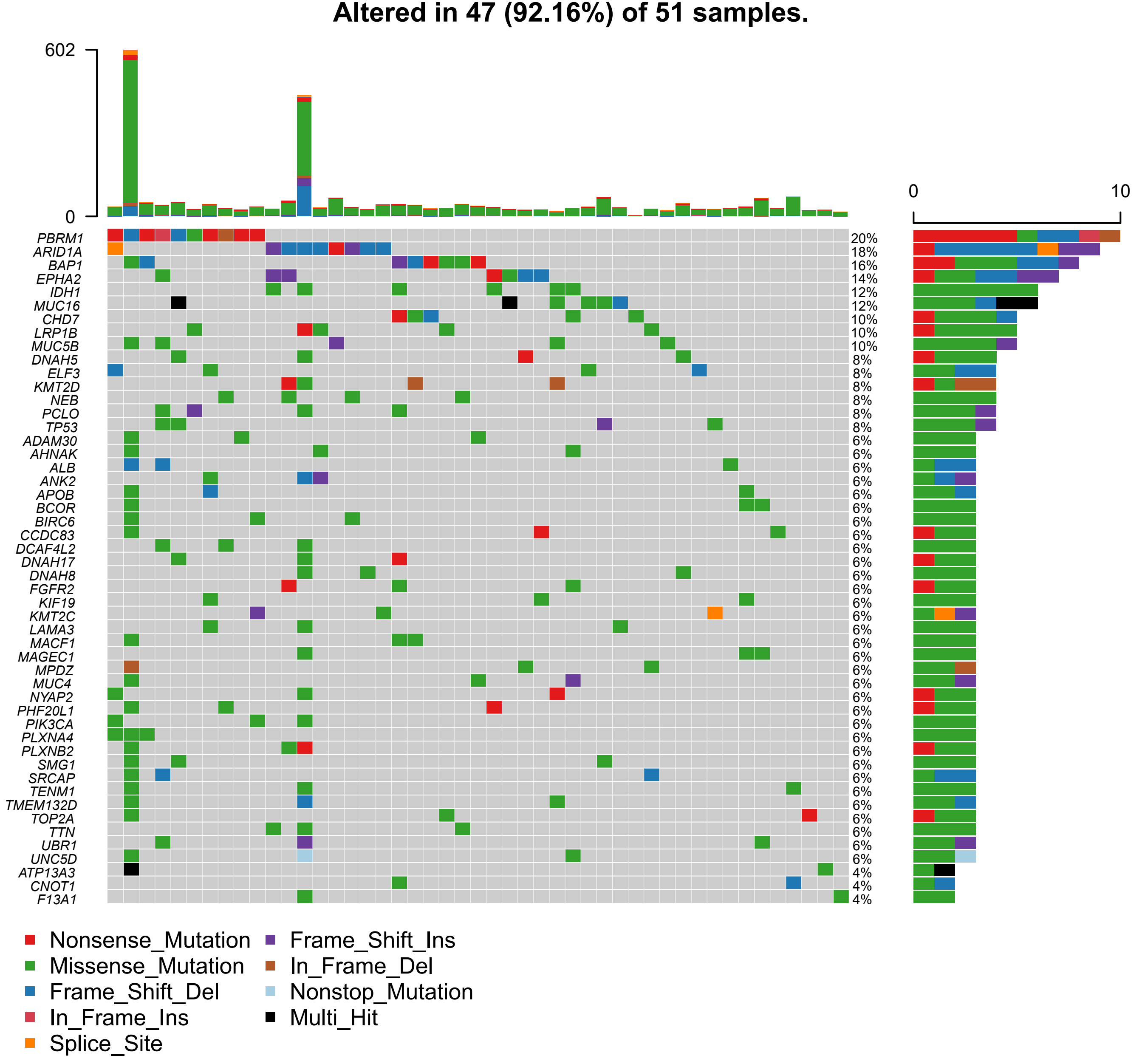
Altered in 47 (92.16%) of 51 samples.
602
0
0
10
PBRM1 20%
ARID1A 18%
BAP1 16%
EPHA2 14%
IDH1 12%
MUC16 12%
CHD7 10%
LRP1B 10%
MUC5B 10%
DNAH5 8%
ELF3 8%
KMT2D 8%
NEB 8%
PCLO 8%
TP53 8%
ADAM30 6%
AHNAK 6%
ALB 6%
ANK2 6%
APOB 6%
BCOR 6%
BIRC6 6%
CCDC83 6%
DCAF4L2 6%
DNAH17 6%
DNAH8 6%
FGFR2 6%
KIF19 6%
KMT2C 6%
LAMA3 6%
MACF1 6%
MAGEC1 6%
MPDZ 6%
MUC4 6%
NYAP2 6%
PHF20L1 6%
PIK3CA 6%
PLXNA4 6%
PLXNB2 6%
SMG1 6%
SRCAP 6%
TENM1 6%
TMEM132D 6%
TOP2A 6%
TTN 6%
UBR1 6%
UNC5D 6%
ATP13A3 4%
CNOT1 4%
F13A1 4%
Nonsense_Mutation
Missense_Mutation
Frame_Shift_Del
In_Frame_Ins
Splice_Site
Frame_Shift_Ins
In_Frame_Del
Nonstop_Mutation
Multi_Hit
